# Extended adjuvant endocrine therapy for women with hormone receptor-positive early breast cancer: A meta-analysis with trial sequential analysis of randomized controlled trials

**DOI:** 10.3389/fonc.2022.1039320

**Published:** 2022-10-27

**Authors:** Ming Xie, Yan Zhong, Yide Yang, Fang Shen, Yue Nie

**Affiliations:** ^1^ Department of Science and Education, The Third Hospital of Changsha, Changsha, China; ^2^ Department of Preventive Medicine, School of Medicine, Hunan Normal University, Changsha, China; ^3^ Key Laboratory of Molecular Epidemiology of Hunan Province, School of Medicine, Hunan Normal University, Changsha, China; ^4^ Department of Geriatrics, The Third Hospital of Changsha, Changsha, China

**Keywords:** aromatase inhibitor, extended adjuvant endocrine therapy, prognosis, disease-free survival, overall survival

## Abstract

**Objectives:**

The aim of the current study is to explore the association between extended adjuvant endocrine treatment and prognosis of women with hormone receptor-positive (HR+) early breast cancer.

**Methods:**

Databases including PubMed, Web of Science, Embase and the Cochrane Library databases were electronically searched to identify randomized controlled trials (RCTs) that reported extended endocrine therapy for women with HR+ early breast cancer. The retrieval time was limited from inception to September 2022. Two reviewers independently screened literature, extracted data, and assessed risk bias of included studies. Meta-analysis was performed by using R software Version 4.1.2 and STATA Version 12.0.

**Results:**

A total of 15 RCTs involving 29497 cases were included. The overall analysis showed that compared with the control, extended adjuvant endocrine therapy increased disease-free survival (DFS) (HR=0.814, 95% CI: 0.720-0.922, 95% PI: 0.556-1.194), overall survival (OS) (HR=0.885, 95% CI: 0.822-0.953, 95% PI: 0.771-1.035), relapse-free survival (RFS) (HR=0.833, 95% CI: 0.747-0.927, 95% PI: 0.575-1.159), distant metastatic-free survival (DMFS) (HR=0.824, 95% CI: 0.694-0.979, 95% PI: 0.300-2.089) and reduced new breast cancer cumulative incidence (NBCCI) (HR=0.484, 95% CI: 0.403-0.583, 95% PI: 0.359-0.654). For adverse events, extended adjuvant endocrine treatment was associated with a significantly higher risk of bone fracture (RR=1.446, 95% CI: 1.208-1.730, 95% PI: 1.154-1.854) and osteoporosis (RR=1.377, 95% CI: 1.018-1.862, 95% PI: 0.347-5.456).

**Conclusion:**

Our study showed that extended adjuvant endocrine therapy increased DFS, OS, RFS, DMFS, the incidence of bone fracture and osteoporosis, and reduced NBCCI.

**Systematic Review Registration:**

https://www.crd.york.ac.uk/prospero, identifier (CRD42022351295)

## Introduction

In recent decades, the treatment of breast cancer has changed substantially with an improved survival over time (1). Over the past 40 years, the risk of dying from breast cancer has fallen by more than 1/3 in the US and Europe, which was attributed to the early detection and improved therapy (2). Adjuvant endocrine therapy (AET) is the foundation of systemic therapy for hormone receptor-positive (HR+) breast cancer patients (3, 4). At present, the standard AET for early breast cancer includes 5-10 years of tamoxifen (TAM), 5-10 years of sequential tamoxifen followed by an aromatase inhibitor (AI), or 5 years of an AI (5–7). Several trials have shown that the risk of late recurrence was reduced after more than 5 years of extended endocrine therapy and prolonged endocrine therapy in patients with early breast cancer has clearly improved patient outcomes (5, 8–10). However, three trials presented at San Antonio Breast Cancer Symposium in 2016 did not show similar benefits for extended adjuvant endocrine therapy beyond 5 years (11–13). Therefore, the research conclusions for extended endocrine therapy are controversial.

Two recently published meta-analyses have reported the effect of extended endocrine therapy on patients with early breast cancer (14, 15). The first meta-analysis revealed that extended endocrine therapy was associated with improvement in breast cancer-specific survival, disease-free survival (DFS), disease recurrence and contralateral breast recurrence. However, the pooled effect of the first meta-analysis was reported as odds ratio (OR) (14). The second meta-analysis showed that prolonged 10-year endocrine treatment improved DFS in patients with early breast cancer (15). Nevertheless, the second study merely reported the outcomes for DFS and overall survival (OS).

Therefore, the objectives of the present study were to assess the the clinical outcomes of extended adjuvant endocrine therapy in women with HR+ early breast cancer by estimating a pooled effect of hazard ratio (HR) extracted from randomized controlled trials (RCTs). In addition, we aimed to supplement the clinical outcomes of relapse-free survival (RFS), distant metastatic-free survival (DMFS), new breast cancer cumulative incidence (NBCCI) and adverse events (AEs), and update DFS and OS of extended endocrine treatment for HR+ early breast cancer.

## Materials and methods

This study does not require ethical approval and informed consent because it is a systematic review and meta-analysis of previously published literature and does not address ethics or patient privacy. Our study was analyzed and reported according to the Preferred Reporting Items for Systematic Reviews and Meta-Analyses (PRISMA) (16). This systematic review protocol has been registered in PROSPERO’s database (registration number: CRD42022351295).

### Search strategy

Two reviewers independently performed a comprehensive literature search in four electronic databases, including PubMed, Web of Science, Embase and the Cochrane Library. All databases were searched through September 2022. The following MeSH terms and keywords were searched: ((breast neoplasm) OR (breast tumor) OR (breast cancer) OR (mammary cancer) OR (breast carcinoma)) AND (hormone OR endocrine OR anti-hormone OR adjuvant OR tamoxifen OR letrozole OR exemestane OR anastrozole OR (aromatase inhibitor)) AND (therapy OR treatment) AND (extend OR extended OR extension OR prolonged OR prolongation) AND ((controlled clinical trial) OR (randomized controlled trial)). The details of the search strategy were provided in **Supplemental Files 1**.

### Inclusion and exclusion criteria

The following criteria were predefined for inclusion of a study: (i) randomized controlled trials (RCTs) comparing the prolonged AET to a control group (placebo, observation or extended treatment for 2-5 years); (ii) the participants were women with HR+ early breast cancer; (iii) hazard ratio (HR) and their 95% confidence intervals (CIs) were reported in the original article or could be extracted from Kaplan-Meier curves. The exclusion criteria were as follows: (i) observational studies; (ii) different endocrine therapy drugs were used in experimental and control arm during extended treatment; (iii) literature reviews, conference abstract, and study protocols.

### Assessment of study quality

We used the modified Jadad scale to assess the quality of RCTs (17). The evaluation criteria of the modified Jadad scale included four items: randomization, randomization concealment, double blind, and withdrawals and dropouts. The score 0-3 out of 7 is considered a low-quality study and a score of 4-7 is a high-quality study. When inconsistency exists, a third reviewer will make the final decision after verification and discussion.

### Data extraction

Screening of studies, selection, exclusion, and data extraction were performed by two reviewers independently. Any disagreements were discussed and reached a consensus. We extracted the following information from RCTs: trial and publication year, RCT type, previous and extended treatment, menopausal state, number and median age of participants, lymph node positive rate, median follow-up, and outcomes (the primary outcomes were DFS and OS, the secondary outcomes were RFS, DMFS, NBCCI and AEs).

### Statistical analysis

Statistical analyses were performed with R software Version 4.1.2 and STATA Version 12.0 (StataCorp, College Station, TX, USA). HR and 95% CI were extracted from Kaplan-Meier curves using Engauge Digitizer version 10.8 (http://markummitchell.github.io/engauge-digitizer/) and methodology by Tierney et al. (18). Data of dichotomous outcomes were pooled using the risk ratio (RR) and presented as the 95% CI. Heterogeneity was assessed statistically by using the Cochran’s Q test, I^2^ and Tau^2^ statistic and 95% prediction interval (PI) (19, 20). When I^2^ < 50%, the results of the associated studies were considered to have acceptable heterogeneity, and a fixed-effects model (using inverse variance method) was utilized. When I^2^ ≥ 50%, it was considered that there was heterogeneity in the results of the included studies, and a random-effects model (using DerSimonian and Laird method) was selected (21). The trim-and-fill method was used to test and adjust for publication bias (22). All the P-values are two-sided.

### Trial sequential analysis

We performed a trial sequential analysis (TSA) to assess if the available evidence is up to the required information size (RIS) for robust conclusion (23). For dichotomous outcomes, the TSA was performed using TSA v0.9.5.10 Beta software (www.ctu.dk/tsa). STATA Version 12.0 (*metacumbounds* and *rsource* function) and R software Version 4.1.2 (*foreign* and *ldbounds* packages) were used to perform TSA for outcomes of DFS, OS, RFS, DMFS and NBCCI with an a priori information size (APIS) method. In the present TSA, we estimated the RIS and built O’Brien-Fleming α-spending boundaries by using type I error of 5% and type II error of 20%, which were two-side values. If the cumulative Z-curve crossed the trial sequential monitoring boundary or RIS boundary, no further trials were considered to be needed and firm evidence was obtained.

## Results

### Procedure of literature selection

**Figure 1:**
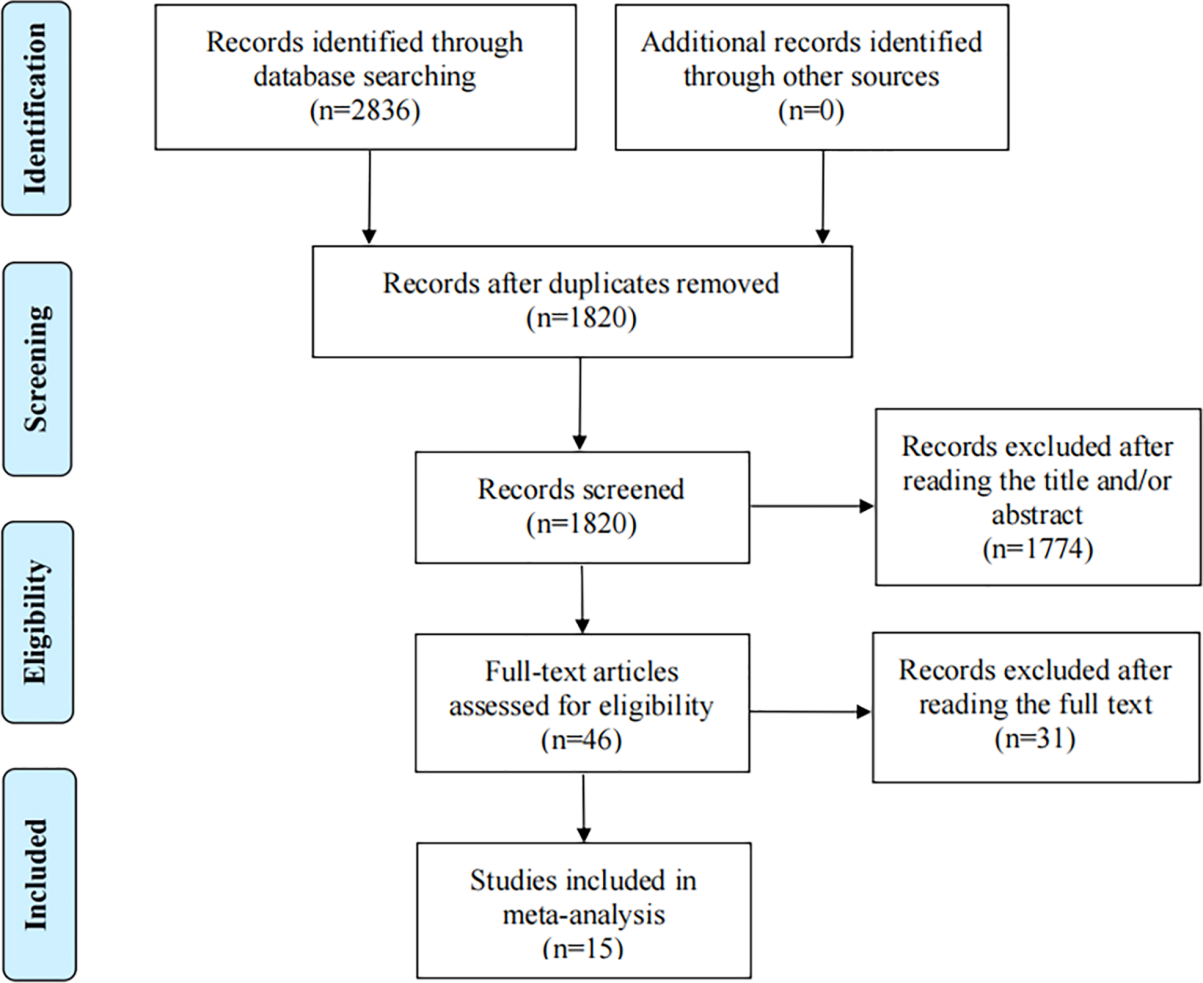
Flow diagram of the process of selection of articles.

The initial search identified 2836 relevant studies (Pubmed: 642, Web of science: 976, Embase: 389, the Cochrane Library: 829). After 1016 duplicate studies were excluded, 1820 articles remained. Then 1774 articles were excluded after screening the titles and abstracts according to the eligibility criteria, eventually, 46 potential articles were reviewed for full-text. After reading the full text, 31 articles did not meet the inclusion criteria: 8 articles did not report Kaplan-Meier curves, HR and their 95%CI; 4 articles were not RCTs of extended endocrine therapy; 19 were conference abstract. Finally, 15 eligible studies (5, 9–11, 24–33) were included in the present meta-analysis (**Figure 1**).

### Characteristics and quality assessment of the included studies

15 RCTs involving 29497 cases were included in our meta-analysis, of which 14 were phase III trials, and one article did not report the trial type. The characteristics of the included trials are shown in **Table 1**. All the studies included scored 4-7, which were considered as high quality, because the study design had been described in detail (**Supplemental Files 2**).

**Table 1:** Characteristics of studies included in the meta-analysis.

Trial (Publication year)	Type	Previous treatment (E/C)	Extended treatment (E/C)	N (E/C)	Menopausal state	Median age (E/C, y)	Lymph node positive (E/C, %)	Median follow-up (y)	Outcomes
NSABP B-33 (2008)	Phase III	TAM 5y/TAM 5y	EXE 5y/Placebo 5y	799/799	Post	NR/NR	48/48	2.5	DFS, RFS, AEs
ECOG (1996)	Phase III	TAM 5y/TAM 5y	TAM 5y/Observation 5y	100/93	Pre/Post	NR/NR	100/100	5.6	RFS, OS
Scottish trial (2001)	NR	TAM 5y/TAM 5y	TAM 5y/Observation 5y	666/656	Pre/Post	NR/NR	NR/NR	15	DFS, OS
NSABP-B14 (2001)	Phase III	TAM 5y/TAM 5y	TAM 5y/Placebo 5y	583/569	Pre/Per	56/56	0/0	6.8	DFS, OS, RFS
ABCSG-6a (2007)	Phase III	TAM 5y/TAM 5y	ANA 3y/Observation 3y	387/469	Post	67.8/68.5	34.1/31.1	5.2	RFS, DMFS, AEs
BOOG 2006-05 (2018)	Phase III	AET 5y/AET 5y	LET 5y/LET 2.5y	915/909	Post	NR/NR	73.3/73.3	6.6	DFS, OS, NBCCI, DMFS, AEs
ABCSG-16 (2021)	Phase III	AET 5y/AET 5y	ANA 5y/ANA 2y	1605/1603	Post	64/64	32.7/33.4	9.8	DFS, OS
IDEAL trial (2018)	Phase III	AET 5y/AET 5y	LET 5y/LET 2.5y	493/499	Post	NR/NR	100/100	7	DFS, OS, DMFS
MA-17R (2016)	Phase III	AI 4.5-6y/AI 4.5-6 y	LET 5y/Placebo 5y	959/959	Post	65.6/64.8	53.5/53.2	6.3	DFS, OS, NBCCI, AEs
ANZ0501 LATER (2016)	Phase III	AET≥4y/AET≥4y	LET 5y/Observation 5y	181/179	Post	65/64	30.9/36.3	3.9	NBCCI, AEs
MA.17 (2008)	Phase III	TAM 4.5-6y/TAM 4.5-6y	LET 5y/Placebo 5y	2583/2587	Post	62.4/62	46/46	5.3	DFS, DMFS, OS, NBCCI
ATLAS (2013)	Phase III	TAM 5y/TAM 5y	TAM 5y/Observation 5y	3428/3418	Pre/Per/Post	NR/NR	43/42	7.6	RFS, OS
DATA (2017)	Phase III	TAM 2-3y+ANA 3y/TAM 2-3y+ANA 3y	ANA 3y/Observation 3y	827/833	Post	NR/NR	67.8/66.3	4.1	DFS, OS, NBCCI, AEs
SCOTTISH (1996)	Phase III	TAM 5y/TAM 5y	TAM 5y/Placebo 5y	173/169	Pre/Post	64/63	24.8/20.7	6.2	EFS
GIM 4 (2021)	Phase III	TAM 2-3y/TAM 2-3y	LET 5y/LET 2-3y	1026/1030	Post	61/60	41.7/39.9	11.7	DFS, OS, AEs

E experimental arm, C control arm, N number of patients, y year, NR not reported, TAM tamoxifen, EXE exemestane, Post postmenopausal, Pre premenopausal, Per perimenopausal, DFS disease-free survival, OS overall survival, RFS relapse-free survival, AEs, adverse events, ANA anastrozole, LET letrozole, AET adjuvant endocrine therapy, AI aromatase inhibitor, DMFS distant metastatic-free survival, NBCCI new breast cancer cumulative incidence.

### DFS and OS of extended endocrine treatment versus the control

**Figure 2:**
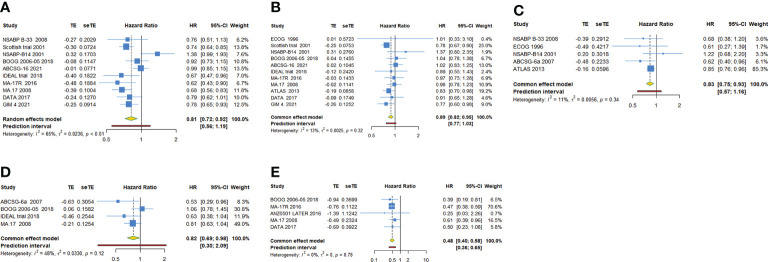
Forest plot of meta-analysis of extended adjuvant endocrine therapy for **(A)** disease-free survival, **(B)** overall survival, **(C)** relapse-free survival, **(D)** distant metastatic-free survival, and **(E)** new breast cancer cumulative incidence.

**Figure 3:**
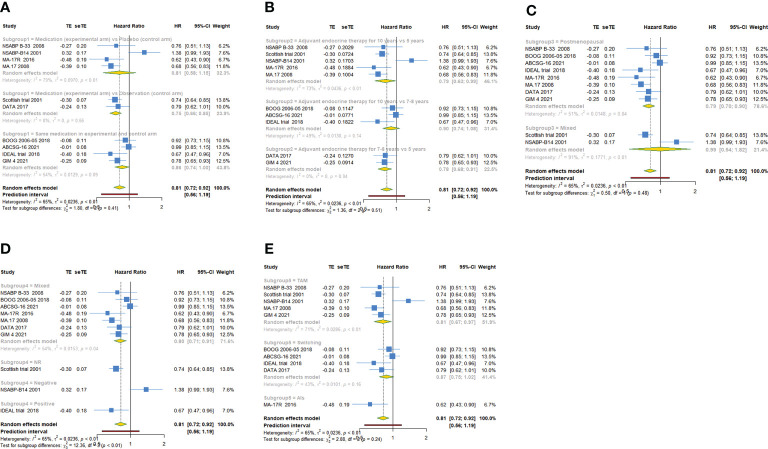
Forest plot of subgroup analysis of extended adjuvant endocrine therapy for disease-free survival (DFS). **(A)** Subgrouped by the extended treatment method in experimental and control arm; **(B)** subgrouped by the duration of adjuvant endocrine therapy; **(C)** subgrouped by menopausal state of patients; **(D)** subgrouped by lymph node positive/negative; **(E)** subgrouped by the type of prior endocrine treatment.

As shown in **Table 2**, 10 trials with a total of 20900 subjects reported DFS. There was medium heterogeneity among the studies concerning DFS, a random-effect model was used to analyze the pooled DFS (I^2^ = 65.0%, Tau^2^ = 0.0236, P=0.002). The overall analysis showed that extended endocrine therapy significantly increased DFS compared with the control (HR=0.814, 95% CI: 0.720-0.922, 95% PI: 0.556-1.194) (**Figure 2A**). The subgroup analysis was undertaken based on the extended treatment method in experimental and control arm (Subgroup 1), the duration of adjuvant endocrine therapy (Subgroup 2), menopausal state of patients (Subgroup 3), lymph node positive/negative (Subgroup 4) and the type of prior endocrine treatment (Subgroup 5). We found that prolonged treatment with drugs increased DFS compared with observation in the control (HR=0.752, 95% CI: 0.665-0.851) and compared with AET for 5 years, AET for 10 years (HR=0.790, 95% CI: 0.632-0.988, 95% PI: 0.371-1.685) or for 7-8 years (HR=0.783, 95% CI: 0.677-0.906) increased DFS. The significant benefit of extended endocrine therapy for DFS was obtained respectively in postmenopausal women (HR=0.793, 95% CI: 0.702-0.895, 95% PI: 0.568-1.107) or lymph node positive/negative patients (HR=0.804, 95% CI: 0.707-0.914, 95% PI: 0.561-1.152). Additionally, we found that extended endocrine treatment increased DFS only in the group that the prior endocrine treatment was TAM (HR=0.811, 95% CI: 0.675-0.974, 95% PI: 0.438-1.500) (**Figure 3**).

**Table 2:** Meta-analysis of DFS for extended versus routinely endocrine treatment in HR+ early breast cancer.

Outcome and subgroups	Number of studies	Number of patients	Meta-analysis				Heterogeneity	
			HR	95% CI	P value	95% PI	I^2^, Tau^2^	P value
DFS	10	20900	0.814	0.720-0.922	0.001	0.556-1.194	65.0%, 0.0236	0.002
Subgrouped by the extended treatment method in experimental and control arm (Subgroup 1)								
Medication (experimental arm) vs Placebo (control arm)	4	9838	0.813	0.575-1.149	0.241	0.174-3.794	79.4%, 0.0970	0.002
Medication (experimental arm) vs Observation (control arm)	2	2982	0.752	0.665-0.851	<0.001	–	0%, 0	0.655
Same medication in experimental and control arm	4	8080	0.860	0.737-1.004	0.057	0.475-1.560	53.6%, 0.0129	0.091
Subgrouped by the duration of adjuvant endocrine therapy (Subgroup 2)								
Adjuvant endocrine therapy for 10 years vs 5 years	5	11160	0.790	0.632-0.988	0.039	0.371-1.685	73.0%, 0.0436	0.005
Adjuvant endocrine therapy for 10 years vs 7-8 years	3	6024	0.896	0.745-1.078	0.243	0.137-5.858	48.8%, 0.0130	0.142
Adjuvant endocrine therapy for 7-8 years vs 5 years	2	3716	0.783	0.677-0.906	0.001	–	0%, 0	0.935
Subgrouped by menopausal state of patients (Subgroup 3)								
Postmenopausal	8	18426	0.793	0.702-0.895	<0.001	0.568-1.107	51.4%, 0.0148	0.045
Mixed (Pre, Per or Post)	2	2474	0.992	0.539-1.824	0.978	–	91.2%, 0.1771	<0.001
Subgrouped by lymph node positive/negative (Subgroup 4)								
Positive	1	992	0.670	0.469-0.958	0.028	–	–	–
Negative	1	1152	1.380	0.988-1.927	0.059	–	–	–
Mixed (positive or negative)	7	17434	0.804	0.707-0.914	<0.001	0.561-1.152	54.4%, 0.0153	0.041
NR	1	1322	0.740	0.642-0.853	<0.001	–	–	–
Subgrouped by the type of prior endocrine treatment (Subgroup 5)								
TAM	5	11298	0.811	0.675-0.974	0.025	0.438-1.500	70.6%, 0.0286	0.009
AIs	1	1918	0.620	0.429-0.897	0.011	–	–	–
Switching (TAM + AIs)	4	7684	0.874	0.751-1.017	0.082	0.506-1.510	42.8%, 0.0101	0.155

DFS, disease-free survival; Pre, premenopausal; Per, perimenopausal; Post, postmenopausal; NR, not reported; TAM, tamoxifen; AI, aromatase inhibitors.

**Figure 4:**
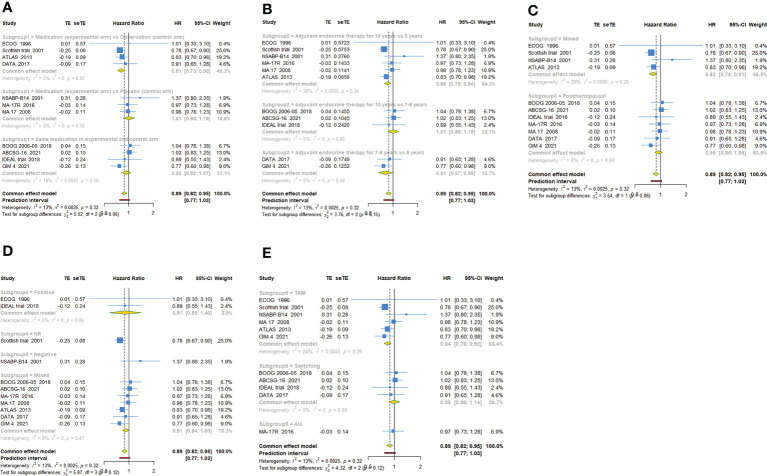
Forest plot of subgroup analysis of extended adjuvant endocrine therapy for overall survival (OS). **(A)** Subgrouped by the extended treatment method in experimental and control arm; **(B)** subgrouped by the duration of adjuvant endocrine therapy; **(C)** subgrouped by menopausal state of patients; **(D)** subgrouped by lymph node positive/negative; **(E)** subgrouped by the type of prior endocrine treatment.

11 trials with a total of 26341 subjects reported OS. There was no significant heterogeneity among the studies concerning OS, a fixed-effect model was used to analyze the pooled OS (I^2 =^ 12.9%, Tau^2^= 0.0025, P=0.321). The overall analysis showed that extended endocrine therapy significantly increased OS compared with the control (HR=0.885, 95% CI: 0.822-0.953, 95% PI: 0.771-1.035) (**Figure 2B**). As shown in **Table 3**, compared with observation in the control, prolonged treatment with drugs increased OS (HR=0.813, 95% CI: 0.732-0.903, 95% PI: 0.645-1.024). In addition, compared with AET for 5 years, AET for 10 years (HR=0.861, 95% CI: 0.785-0.944, 95% PI: 0.675-1.148) or for 7-8 years (HR=0.814, 95% CI: 0.668-0.994) increased OS. The significant benefit of extended endocrine treatment for OS was obtained in the group that the prior endocrine treatment was TAM (HR=0.837, 95% CI: 0.765-0.917, 95% PI: 0.664-1.083). No significant benefit of extended endocrine therapy for OS was observed in postmenopausal women (HR=0.946, 95% CI: 0.855-1.046, 95% PI: 0.829-1.079) or lymph node positive patients (HR=0.907, 95% CI: 0.586-1.404) (**Figure 4**).

**Table 3:** Meta-analysis of OS for extended versus routinely endocrine treatment in HR+ early breast cancer.

Outcome and subgroups	Number of studies	Number of patients	Meta-analysis				Heterogeneity	
			HR	95% CI	P value	95% PI	I^2^, Tau^2^	P value
OS	11	26341	0.885	0.822-0.953	0.001	0.771-1.035	12.9%, 0.0025	0.321
Subgrouped by the extended treatment method in experimental and control arm (Subgroup 1)								
Medication (experimental arm) vs Placebo (control arm)	3	8240	1.008	0.853-1.191	0.926	0.343-2.965	0%, 0	0.504
Medication (experimental arm) vs Observation (control arm)	4	10021	0.813	0.732-0.903	<0.001	0.645-1.024	0%, 0	0.821
Same medication in experimental and control arm	4	8080	0.934	0.818-1.066	0.311	0.601-1.444	18.4%, 0.0045	0.299
Subgrouped by the duration of adjuvant endocrine therapy duration (Subgroup 2)								
Adjuvant endocrine therapy for 10 years vs 5 years	6	16601	0.861	0.785-0.944	0.001	0.675-1.148	26.4%, 0.0055	0.236
Adjuvant endocrine therapy for 10 years vs 7-8 years	3	6024	1.011	0.864-1.183	0.893	0.365-2.796	0%, 0	0.851
Adjuvant endocrine therapy for 7-8 years vs 5 years	2	3716	0.814	0.668-0.994	0.044	–	0%, 0	0.437
Subgrouped by menopausal state of patients (Subgroup 3)								
Postmenopausal	7	16828	0.946	0.855-1.046	0.277	0.829-1.079	0%, 0	0.692
Mixed (Pre, Per or Post)	4	9513	0.821	0.736-0.914	<0.001	0.526-1.330	26.0%, 0.0060	0.256
Subgrouped by lymph node positive/negative (Subgroup 4)								
Positive	2	1185	0.907	0.586-1.404	0.662	–	0%, 0	0.839
Negative	1	1152	1.370	0.798-2.353	0.254	–	–	–
Mixed (positive or negative)	7	22682	0.914	0.837-0.999	0.046	0.815-1.026	0%, 0	0.473
NR	1	1322	0.780	0.673-0.904	0.001	–	–	–
Subgrouped by the type of prior endocrine treatment (Subgroup 5)								
TAM	6	16739	0.837	0.765-0.917	<0.001	0.664-1.083	23.5%, 0.0045	0.257
AIs	1	1918	0.970	0.733-1.284	0.832	–	–	–
Switching (TAM + AIs)	4	7684	0.993	0.861-1.145	0.918	0.726-1.358	0%, 0	0.892

OS, overall survival; Pre, premenopausal; Per, perimenopausal; Post, postmenopausal; NR, not reported; TAM, tamoxifen; AIs, aromatase inhibitors.

### RFS, DMFS and NBCCI of extended endocrine treatment versus the control

For RFS, five trials with a total of 10645 subjects reported the HR and 95%CI. Our analysis with a fixed-effect model showed that extended adjuvant endocrine therapy was associated with an increased RFS (HR=0.833, 95% CI: 0.747-0.927, 95% PI: 0.575-1.159, I^2^= 11.0%, Tau^2^= 0.0056) (**Table 4**, **Figure 2C**). Four trials involving 8842 participants reported DMFS. Our analysis with a fixed-effect model observed that extended endocrine treatment significantly increased DMFS (HR=0.824, 95% CI: 0.694-0.979, 95% PI: 0.300-2.089, I^2 =^ 47.9%, Tau^2 =^ 0.0330) (**Table 4**; **Figure 2D**). For NBCCI, five trials with a total of 10932 subjects were included in our analysis. The result with a fixed-effect model showed that extended adjuvant endocrine therapy reduced NBCCI (HR=0.484, 95% CI: 0.403-0.583, 95% PI: 0.359-0.654, I^2 =^ 0, Tau^2 =^ 0) (**Table 4**; **Figure 2E**).

**Table 4:** Meta-analysis of RFS, DMFS, NBCCI and AEs for extended versus routinely endocrine treatment in HR+ early breast cancer.

Outcome and subgroups	Number of studies	Number of patients	Meta-analysis				Heterogeneity	
			HR/RR	95% CI	P value	95% PI	I^2^, Tau^2^	P value
RFS	5	10645	0.833	0.747-0.927	0.001	0.575-1.159	11.0%, 0.0056	0.343
DMFS	4	8842	0.824	0.694-0.979	0.028	0.300-2.089	47.9%, 0.0330	0.124
NBCCI	5	10932	0.484	0.403-0.583	<0.001	0.359-0.654	0%, 0	0.781
AEs								
Hot flashes	4	6158	1.088	0.994-1.190	0.065	0.724-1.722	38.3%, 0.0062	0.182
Bone fracture	7	10272	1.446	1.208-1.730	<0.001	1.154-1.854	0%, 0	0.536
Osteoporosis	4	5762	1.377	1.018-1.862	0.038	0.347-5.456	84.7%, 0.0787	<0.001
Arthralgia	4	6158	1.041	0.907-1.195	0.569	0.575-1.886	74.0%, 0.0141	0.009

RFS relapse-free survival, DMFS distant metastatic-free survival, NBCCI new breast cancer cumulative incidence, AEs adverse events.

### EFS of extended endocrine treatment versus the control

For EFS, the trial SCOTTISH reported that extended TAM therapy for 5 years had no effect on EFS (HR=1.270, 95%CI: 0.871-1.852). Since only 1 article reported the effect of extended adjuvant endocrine therapy on prognosis of EFS, it cannot be performed with a meta-analysis in our study.

### Adverse events of extended endocrine treatment versus the control

**Figure 5:**
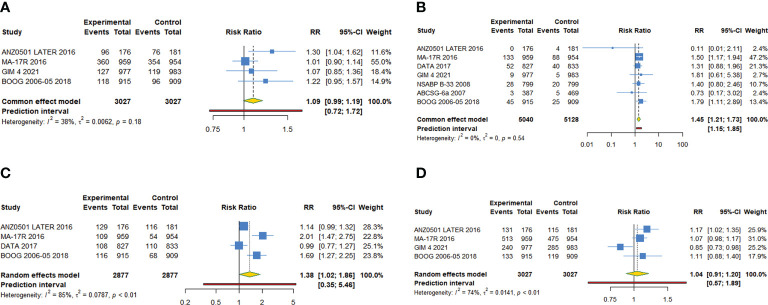
Forest plot of meta-analysis of extended adjuvant endocrine therapy for adverse events. **(A)** Hot flashes, **(B)** bone fracture, **(C)** osteoporosis, **(D)** arthralgia.

Four studies reported hot flashes, with no statistical heterogeneity between the included studies (I^2^ = 38.3%, Tau^2^ = 0.0062, P=0.182). A fixed-effect model was used for statistical analysis. Our meta-analysis revealed that extended adjuvant endocrine therapy group was without a significant risk of hot flashes (RR=1.088, 95% CI: 0.994-1.190, 95% PI: 0.724-1.722) compared with routine group (**Table 4**; **Figure 5A**).

Seven studies reported bone fracture, revealing that extended treatment group was associated with a significantly higher risk of bone fracture (RR=1.446, 95% CI: 1.208-1.730, 95% PI: 1.154-1.854) compared with control group (**Table 4**; **Figure 5B**). There was no statistical heterogeneity between the included studies (I^2^ = 0%, Tau^2^ = 0, P=0.536).

Four studies reported osteoporosis, revealing that extended adjuvant endocrine treatment was associated with a significantly higher risk of osteoporosis (RR=1.377, 95% CI: 1.018-1.862, 95% PI: 0.347-5.456) compared with the control (**Table 4**; **Figure 5C**). There was significant heterogeneity between the included studies (I^2^ = 84.7%, Tau^2^ = 0.0787, P<0.001).

Four studies reported arthralgia, revealing that no risk difference (RR=1.041, 95% CI: 0.907-1.195, 95% PI: 0.575-1.886) between extended and routinely endocrine treatment (**Table 4**; **Figure 5D**). There was significant heterogeneity between the included studies (I^2^ = 74.0%, Tau^2^ = 0.0141, P=0.009).

### Trial sequential analysis results

In trial sequential analysis of DFS, OS, RFS, DMFS and NBCCI, we estimated a required sample size of 1990 (APIS=1990), and the cumulative Z-curve significantly crossed the conventional monitoring boundary and RIS boundary, further suggesting that no additional studies were needed for a stable conclusion (**Supplemental Files 3**; **Figure S1**). For adverse events, trial sequential analysis estimated a required information size of 28854, 10890 and 13025 respectively for hot flashes, osteoporosis and arthralgia, which greatly exceeded the accumulated sample size in our study. Furthermore, although accumulative Z-curve crossed conventional monitoring boundary, it did not cross the trial sequential monitoring boundary. Therefore, we cannot draw a definitive conclusion about hot flashes, osteoporosis and arthralgia due to the presence of false positive. However, a relatively definite conclusion of bone fracture can be obtained from this meta-analysis, as the the cumulative Z-curve crossed both the conventional monitoring boundary and RIS boundary (**Supplemental Files 3**; **Figure S2**).

### Sensitivity analysis and publication bias

We performed a sensitivity analysis for DFS and OS by calculating the pooled HRs and the corresponding 95% CIs after individual studies were omitted to assess whether the pooled results were affected by a single study. The removal of any single study had no significant effect on the quantitative results, suggesting that the pooled results were robust and reliable. The results were shown in **Figure S3** (**Supplementary Files 3**). Trim-and-fill analysis was conducted for DFS and OS, and a funnel plot with imputed studies was obtained in OS, suggesting the existence of publication bias. After the trim-and-fill analysis, the correction for potential publication bias did not alter the result of OS. The funnel plots of trim-and-fill method were shown in **Figure S4** (**Supplementary Files 3**).

## Discussion

Due to the role of estrogen receptor (ER) in the biology of breast cancer, modulation of estrogen signal through endocrine therapy has long been an important component of the treatment for all stages of HR+ breast cancer (34). HR+ breast cancer accounts for an estimated 75% of breast cancer patients, and therefor early breast cancer can benefit from endocrine treatment (35). Standard endocrine therapy consists of daily oral anti-estrogens for five consecutive years, with different treatment options according to menopausal status. Tamoxifen is a selective ER modulator, which competitively inhibits the binding of estrogen and ER and is effective for premenopausal and postmenopausal women (36). AIs (anastrozole, exemestane, and letrozole) reduce circulating estrogen levels by inhibiting androgen to estrogen conversion and are effective only in postmenopausal women (including those in postmenopausal women due to medical ovarian suppression or oophorectomy) (37). The current 5- or 10-year AET for early breast cancer is based on the early results of tamoxifen adjuvant therapy (5, 38). However, it was reported that about 50% of breast cancer recurrences happened after the initial 5-years adjuvant treatment (39). These results initiated a debate about the extended adjuvant endocrine therapy, and numerous studies was carried out to elucidate on this matter.

The present meta-analysis compared the extended adjuvant endocrine therapy and non-extended standard endocrine therapy for HR+ early breast cancer. Our results demonstrated the benefits of extended endocrine therapy for DFS, OS, RFS, DMFS and NBCCI in patients with HR+ early breast cancer and showed that extended treatment group was associated with a significantly higher risk of bone fracture and osteoporosis compared with control group. Subgroup analysis revealed that AET for 10 years or 7-8 years increased DFS and OS compared with AET for 5 years, the significant benefit of extended endocrine therapy for DFS was obtained in postmenopausal women, and extended adjuvant endocrine treatment increased DFS and OS only in the group that the prior endocrine treatment was TAM.

Whether prolonged AET can further improve the clinical benefit has been a controversial topic. Several large clinical trials have shown that AET for 5 years significantly decreases the risks of breast cancer death, local and distant recurrence, contralateral breast cancer and death from any cause (7, 38, 40). The clinical application of extended endocrine treatment should be carefully weighed against the differences in study population and background in different trials (15). Our results showed that extended adjuvant endocrine therapy increased DFS and OS in patients with hormone receptor-positive early breast cancer. A recent meta-analysis demonstrated that compared with routinely 5-year therapy, extended adjuvant endocrine therapy for 10 years had no effect on OS, which was inconsistent with our result. The causes for the inconsistence may be the differences in studies included in the two meta-analysis and difference in the follow-up duration between included studies (15). For the trial GIM 4 with a median follow-up of 11.7 years included in our study, 15y-DFS was reported with a significant difference (HR=0.78, 95% CI: 0.65-0.93), while 5y-DFS extracted from Kaplan-Meier curves showed no significant difference (HR=1.05, 95%CI: 0.67-1.65). Indeed, the endocrine therapy has a carryover effect with an increase in absolute survival benefit over time, which becomes very pronounced in the second decade after diagnosis compared with the first 5 years of follow-up (7, 8). It becomes therefore obvious that an adequate follow-up duration is crucial in the attempt to assess the benefit of extended adjuvant endocrine treatment (41). Moreover, since OS is the result of breast cancer-related and non-related events, it could be considered that statistically significant OS differences are difficult to be obtained in the presence of a small number of events. Events unrelated to disease recurrence may overtake the ones related to early breast cancer morbidity and mortality, thus masking the actual experimental therapeutic benefits (42). We speculated that the benefit of extended adjuvant endocrine therapy for DFS and OS in early breast cancer patients was more pronounced over time. More RCTs with longer follow-up duration were required to verify our hypothesis.

Additionally, our meta-analysis showed a NBCCI reduction of extending endocrine treatment. The result was consistent with the MA.17R trial, in which the majority of the effect of 5-year LET was explained as prevention of contralateral breast cancer (10). It could be argued that prolonged AI adjuvant therapy for 5-10 years has a significant effect on preventing the recurrence of breast cancer (43, 44). The benefits of extended endocrine therapy for RFS and DFMS were also obtained in our analysis. Evidence from the ATLAS trial existed that 10-year tamoxifen in estrogen receptor-positive breast cancer significantly reduced the recurrence rate and mortality rate of breast cancer not only during the first decade of continuous treatment but also during the second decade after the end of treatment (5). The mechanism of extended adjuvant endocrine therapy to reduce DMFS has not been clear. It may be that mutations in the gene encoding for estrogen receptor are associated with resistance against aromatase inhibitors (34, 45). Dormant tumor cells may become less resistant to AIs, causing the extended therapy to have significant benefit. Nevertheless, our study was still unable to determine the optimal duration of prolonged treatment. For the IDEAL trial and ABCSG-16, patients receiving sequential TAM and AIs or TAM alone or AIs alone for an initial 5 years were randomly assigned to receive 5-year extended AIs treatment (the total duration of AET was 10 years) and 2-2.5 years of prolonged AIs treatment (the total duration of AET was 7-7.5 years). Both trials showed that AET for 10 years was not superior to 7-7.5 years. Subgroup analysis showed that AET for 10 years or 7-8 years improved DFS and OS compared with AET for 5 years. We cannot conclude that the 10-year benefit of adjuvant endocrine treatment is superior to that of 7-8 years by comparing the size of HR values (DFS: 10 years: HR=0.790, 7-8 years: HR=0.783; OS: 10 years: HR=0.861, 7-8 years: HR=0.814).

Our analysis revealed that extended adjuvant endocrine treatment was associated with a significantly higher risk of bone fracture and osteoporosis. The adverse events and clinical efficacy are two decisive factors for extended adjuvant endocrine treatment. The persistence of endocrine therapy can be interrupted by serious adverse events (46, 47). Thus, accurate assessment of adverse events caused by extended treatment is critical to smooth implementation of the trial. There was sufficient evidence that the use of AIs increased the risk of bone related adverse events, such as bone loss and fracture rate (48). The administration of AI can inhibit the conversion of androgens to estrogens, leading to osteopenia and osteoporosis (49, 50). This may cause an increase in the incidence of fragility fractures among patients taking AI (51, 52). A mouse model study of breast cancer demonstrated that AIs, used as blockers of estrogen biosynthesis for standard endocrine therapy, can cause muscle weakness and bone loss (53).

There were some important limitations in our work. First, some of the clinical outcomes (DFS, OS or RFS, etc) were extracted from the Kaplan-Meier curves, which may cause accidental error for HR value and their 95% CI and mask some statistically significant results. Second, different trials have different follow-up times, and this limitation may not capture some late deaths or recurrences that are commonly observed in this disease. Third, due to the differences of outcome definition between included trials (**Supplemental Files 4**), uncorrectable heterogeneity existed in present analysis (e.g., RFS in MA.17R included only recurrences of original breast cancer or a new breast cancer while NSABP-B33 included also new non-breast primary cancer and death from any cause). Fourth, the TSA results of hot flashes, osteoporosis and arthralgia showed that the cumulative Z-curve crossed neither the the trial sequential monitoring boundary nor RIS boundary, suggesting that more high-quality RCTs with large sample size were needed in future research.

## Conclusion

In summary, our meta-analysis demonstrated the benefits of extended adjuvant endocrine therapy for DFS, OS, RFS, DMFS and NBCCI in women with HR+ early breast cancer and showed that extended treatment group was associated with a significantly higher risk of bone fracture and osteoporosis compared with control group.

## Data availability statement

The original contributions presented in the study are included in the article/**Supplementary Material**. Further inquiries can be directed to the corresponding author.

## Author contributions

MX and YN conceived and participated in the design of this review. YZ and MX performed the literature searches, study selection, data extraction and assessed the risk of bias. MX and YZ drafted the manuscript. YY and FS helped in performing the analysis with constructive discussions. YY and YN revised the final version. All authors have made substantial contributions to this work and have approved the final version of the manuscript.

## Funding

This work was supported by the Scientific Research Project of Health Commission of Changsha (2017) and Construction Project of Clinical Teaching Base of Central South University (2020, 2021).

## Conflict of interest

The authors declare that the research was conducted in the absence of any commercial or financial relationships that could be construed as a potential conflict of interest.

## Publisher’s note

All claims expressed in this article are solely those of the authors and do not necessarily represent those of their affiliated organizations, or those of the publisher, the editors and the reviewers. Any product that may be evaluated in this article, or claim that may be made by its manufacturer, is not guaranteed or endorsed by the publisher.
