# Supplementary material for: Extended adjuvant endocrine therapy for women with hormone receptor-positive early breast cancer: A meta-analysis with trial sequential analysis of randomized controlled trials
Source: Front Oncol. 2022 Oct 27;12:1039320. doi: 10.3389/fonc.2022.1039320 (PMC9647050; doi:10.3389/fonc.2022.1039320)
Supplement: Supplementary file 1 [file Table_1.docx]

| **TABLE S1** Quality analysis of each study by modified Jadad scale | | | | | | |
| --- | --- | --- | --- | --- | --- | --- |
| Study | Randomization | Randomization concealment | Double blind | Withdrawals and dropouts | Score | Study quality |
| NSABP B-33 (2008) | 2 | 1 | 2 | 1 | 6 | High |
| ECOG (1996) | 2 | 2 | 0 | 1 | 5 | High |
| Scottish trial (2001) | 2 | 2 | 0 | 1 | 5 | High |
| NSABP-B14 (2001) | 2 | 1 | 2 | 1 | 6 | High |
| ABCSG-6a (2007) | 2 | 1 | 0 | 1 | 4 | High |
| BOOG 2006-05 (2018) | 2 | 2 | 0 | 1 | 5 | High |
| ABCSG-16 (2021) | 2 | 2 | 0 | 1 | 5 | High |
| IDEAL trial (2018) | 2 | 2 | 0 | 1 | 5 | High |
| MA-17R (2016) | 2 | 2 | 2 | 1 | 7 | High |
| ANZ0501 LATER (2016) | 2 | 2 | 0 | 1 | 5 | High |
| MA.17 (2008) | 2 | 2 | 2 | 1 | 7 | High |
| ATLAS (2013) | 2 | 2 | 0 | 1 | 4 | High |
| DATA (2017) | 2 | 2 | 0 | 1 | 5 | High |
| SCOTTISH (1996) | 2 | 2 | 0 | 1 | 5 | High |
| GIM 4 (2021) | 2 | 2 | 0 | 1 | 5 | High |
