# Supplementary material for: Extended adjuvant endocrine therapy for women with hormone receptor-positive early breast cancer: A meta-analysis with trial sequential analysis of randomized controlled trials
Source: Front Oncol. 2022 Oct 27;12:1039320. doi: 10.3389/fonc.2022.1039320 (PMC9647050; doi:10.3389/fonc.2022.1039320)
Supplement: Supplementary file 2 [file Table_2.docx]

| **TABLE S2** Outcome definition of studies included in the meta-analysis. | | |
| --- | --- | --- |
| Trial (Publication year) | Outcomes | Definition |
| NSABP B-33 (2008) | DFS | Recurrence in the chest wall after mastectomy or in the ipsilateral breast after lumpectomy, regional and distant recurrence, second primary cancer (other than squamous or basal cell carcinoma of the skin, carcinoma in situ of the cervix, or lobular carcinoma in situ of the breast), and death resulting from any cause before recurrence or second primary. |
|  | RFS | Time to recurrence or contralateral breast cancer. Other second primary cancers and deaths without evidence of disease were treated as censored events for RFS. |
| ECOG (1996) | RFS | Analyses of the RFS used the date of the first documented evidence of relapse, judged by previously published guidelines, as the date of relapse and included new opposite-breast primary tumors as treatment failures; patients who died without disease recurrence were censored in these analyses at the date of their death. |
|  | OS | Deaths from all causes. |
| Scottish trial (2001) | DFS | Survival free of systemic (including contralateral) disease. |
|  | OS | Deaths from all causes. |
| NSABP-B14 (2001) | DFS | The events used in the determination of DFS included first local recurrence of disease (including ipsilateral breast tumor recurrence after lumpectomy), regional and distant metastases, occurrence of tumor in the contralateral breast, occurrence of second primary tumors, and deaths before these events. |
|  | OS | Deaths from all causes. |
|  | RFS | Events for determination of RFS included a first recurrence of disease at local, regional, or distant sites, but not included contralateral breast cancers. |
| ABCSG-6a (2007) | RFS | The interval between the start of anastrozole treatment or of the observation period (for the no further treatment group) and the first evidence of locoregional recurrence, contralateral breast cancer, or distant metastasis. |
|  | DMFS | Distant metastases included all distant lymph node recurrences (i.e., supraclavicular, mammaria internal, and contralateral axilla) and organ metastases. |
| BOOG 2006-05 (2018) | DFS | The time from random assignment to recurrence (either local, regional, or distant), new primary breast tumors (ductal carcinoma in situ or invasive) or death due to any cause, whichever came first. Second primary non–breast cancer was not included in the definition of DFS. |
|  | OS | Deaths from all causes. |
|  | DMFS | Distant metastasis-free interval. |
|  | NBCCI | New primary breast malignancies (contralateral or new ipsilateral breast cancer). |
| ABCSG-16 (2021) | DFS | Freedom from local or distant metastases, contralateral breast cancer, second primary cancer, or death without recurrence, evaluated in a time-to-event analysis. |
|  | OS | Deaths from all causes. |
| IDEAL trial (2018) | DFS | The time from randomization to recurrence (either local, regional, or distant), new primary breast tumors (ductal carcinoma in situ or invasive) or death due to any cause at 2.5 years after randomization. |
|  | OS | Time to death due to any cause starting at 2.5 years after randomization. |
|  | DMFS | Time to distant recurrence starting at 2.5 years after randomization. |
| MA-17R (2016) | DFS | The time from randomization to recurrence of breast cancer (in the breast or chest wall or at nodal or metastatic sites) or the development of a new primary breast cancer. |
|  | OS | Deaths from all causes. |
|  | NBCCI | Cumulative incidence of contralateral breast cancer. |
| ANZ0501 LATER (2016) | NBCCI | New invasive primary, local, regional, or distant recurrence, or contralateral breast cancer. |
| MA.17 (2008) | DFS | DFS included any breast cancer recurrence and contralateral breast cancer as events. |
|  | OS | Death due to any cause as an event. |
|  | DMFS | DMFS had distant metastasis as the only event. |
|  | NBCCI | Cumulative incident of contralateral breast cancers. |
| ATLAS (2013) | OS | Deaths from all causes. |
|  | RFS | Recurrence (censored at death from other causes). |
| DATA (2017) | DFS | The disease-free survival beyond 3 years after randomisation. Events ending a period of disease-free survival included non-invasive and invasive breast cancer recurrences (local, regional, or distant), second primary non-invasive and invasive breast and other cancers other than basal-cell or squamous-cell carcinoma of the skin and carcinoma in situ of the cervix, and death from any cause. |
|  | OS | Deaths from all causes beyond 3 years after randomisation. |
|  | NBCCI | Cumulative incidence of secondary breast cancer. |
| SCOTTISH (1996) | EFS | Event-free survival (for relapse or death without relapse). |
| GIM 4 (2021) | DFS | DFS was computed from the date of random assignment to one of the following events: local recurrence, distant metastasis, contralateral or ipsilateral breast tumour (excluding ductal carcinoma in situ), second primary malignancy, death from any cause, loss to follow-up, or end of study, whichever occurred first. |
|  | OS | OS was computed from the date of random assignment to the date of death from any cause, loss to follow-up, or end of study. |
| DFS disease-free survival, RFS relapse-free survival, OS overall survival, DMFS distant metastatic-free survival, NBCCI new breast cancer cumulative incidence. | | |
