# Supplementary material for: Extended adjuvant endocrine therapy for women with hormone receptor-positive early breast cancer: A meta-analysis with trial sequential analysis of randomized controlled trials
Source: Front Oncol. 2022 Oct 27;12:1039320. doi: 10.3389/fonc.2022.1039320 (PMC9647050; doi:10.3389/fonc.2022.1039320)
Supplement: Supplementary file 3 [file DataSheet_1.docx]

**FIGURE S1 |** Trial sequential analysis (TSA) between the extended adjuvant endocrine treatment and (A) disease-free survival, (B) overall survival, (C) relapse-free survival, (D) distant metastatic-free survival, and (E) new breast cancer cumulative incidence. α = 5%, β = 20% and relative risk reduction (RRR) = 15%.


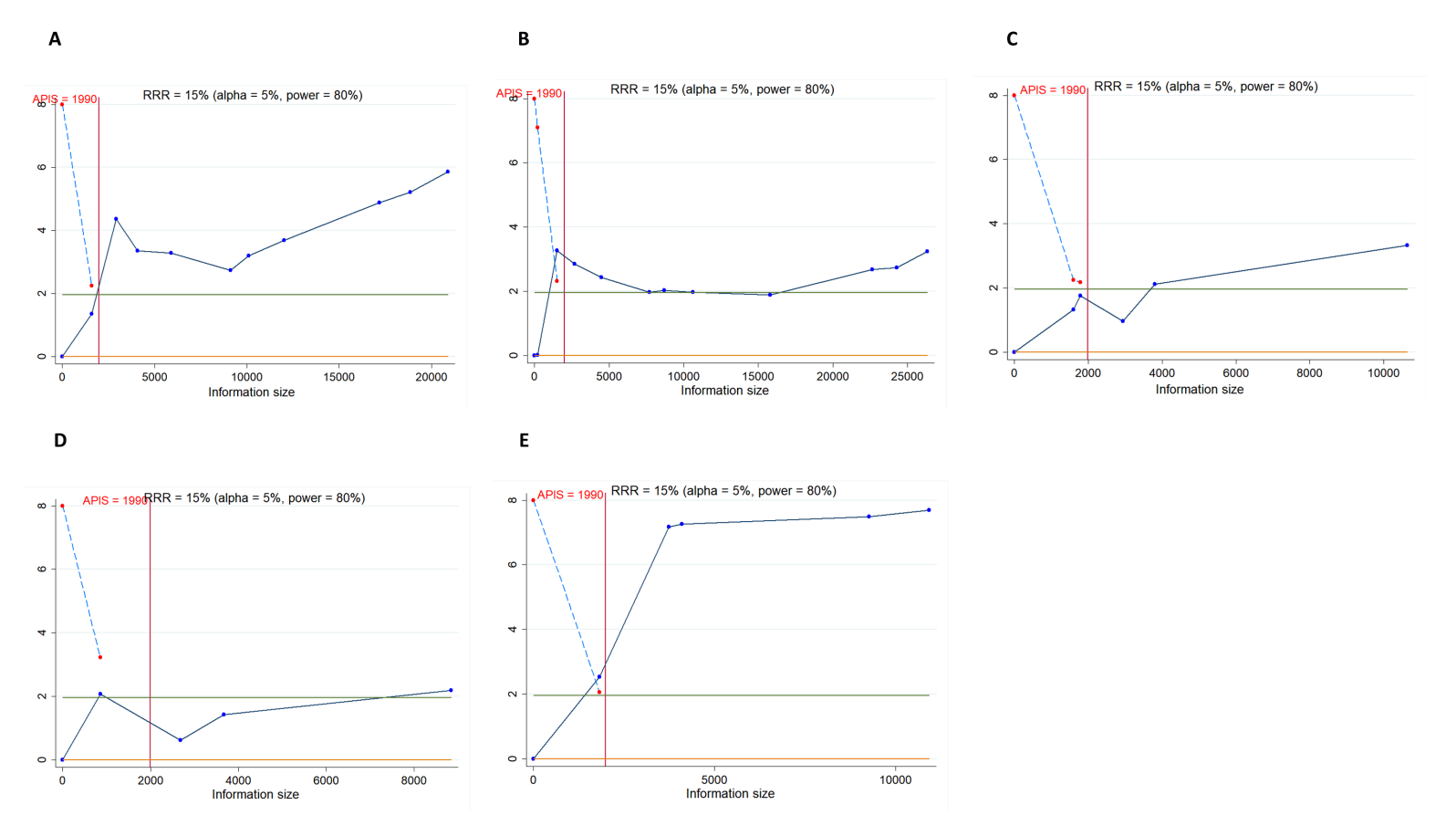


**FIGURE S2 |** Trial sequential analysis (TSA) between the extended adjuvant endocrine treatment and adverse events. (A) Hot flashes, (B) bone fracture, (C) osteoporosis, (D) arthralgia. Uppermost and lowermost curves represent trial sequential monitoring boundary lines for benefit and harm, respectively. Horizontal lines represent the conventional boundaries for statistical significance. Triangular lines represent the futility boundary.


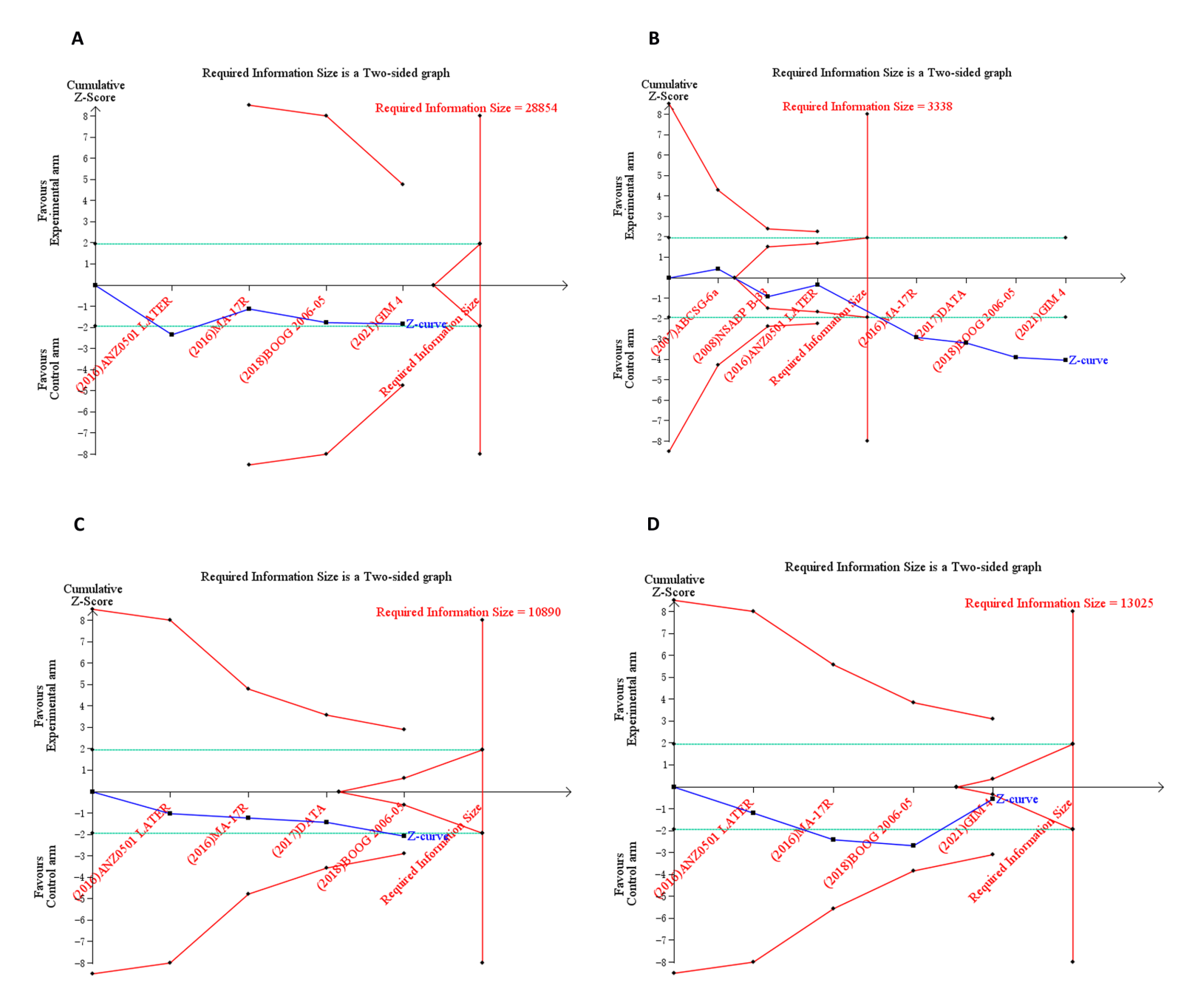


**FIGURE S3 |** Sensitivity analysis on extended adjuvant endocrine therapy for (A) disease-free survival and (B) overall survival.


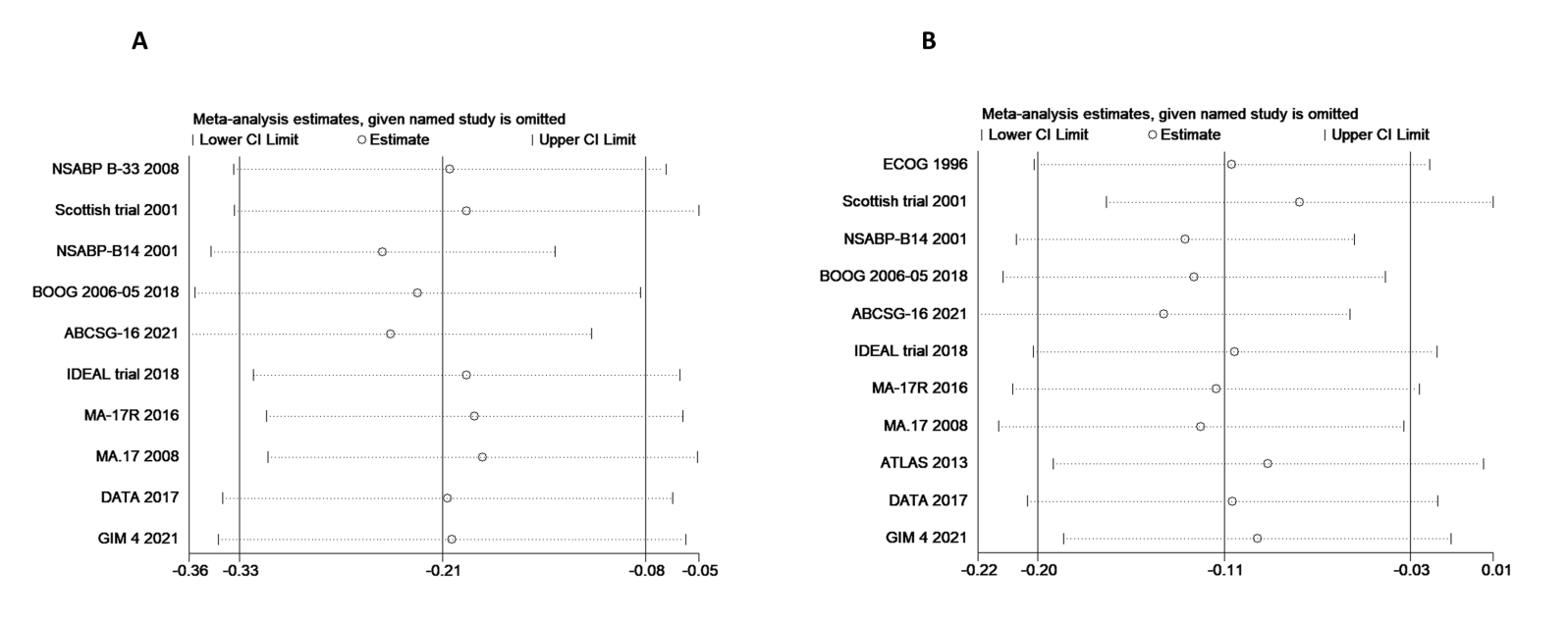

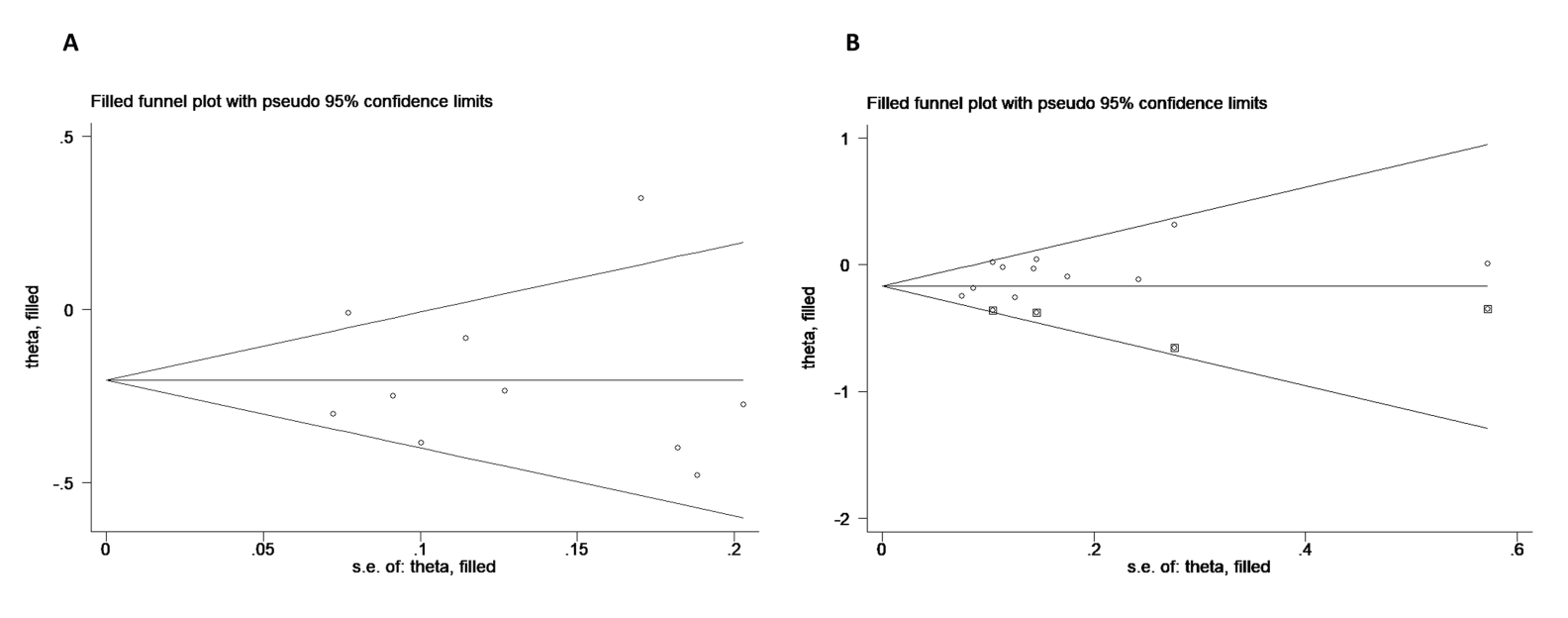


**FIGURE S4 |** Funnel plot of trim-and-fill method on extended adjuvant endocrine therapy for (A) disease-free survival and (B) overall survival.
