# Supplementary material for: Extended adjuvant endocrine therapy for women with hormone receptor-positive early breast cancer: A meta-analysis with trial sequential analysis of randomized controlled trials
Source: Front Oncol. 2022 Oct 27;12:1039320. doi: 10.3389/fonc.2022.1039320 (PMC9647050; doi:10.3389/fonc.2022.1039320)
Supplement: Supplementary file 4 [file DataSheet_2.docx]

Pubmed 642

((breast neoplasm) OR (breast tumor) OR (breast cancer) OR (mammary cancer) OR (breast carcinoma)) AND (hormone OR endocrine OR anti-hormone OR adjuvant OR tamoxifen OR letrozole OR exemestane OR anastrozole OR (aromatase inhibitor)) AND (therapy OR treatment) AND (extend OR extended OR extension OR prolonged OR prolongation) AND ((controlled clinical trial) OR (randomized controlled trial))

Web of Science 976

(TS=(breast neoplasm) OR TS=(breast tumor) OR TS=(breast cancer) OR TS=(mammary cancer) OR TS=(breast carcinoma)) AND (TS=hormone OR TS=endocrine OR TS=anti-hormone OR TS=adjuvant OR TS=tamoxifen OR TS=letrozole OR TS=exemestane OR TS=anastrozole OR TS=(aromatase inhibitor)) AND (TS=therapy OR TS=treatment) AND (TS=extend OR TS=extended OR TS=extension OR TS=prolonged OR TS=prolongation) AND (TS=(controlled clinical trial) OR TS=(randomized controlled trial))

Embase 389

#1 'breast neoplasm'/exp OR 'breast tumor'/exp OR 'breast cancer'/exp OR 'mammary cancer'/exp OR 'breast carcinoma'/exp 634335

#2 'hormone'/exp OR endocrine OR 'anti hormone' OR 'adjuvant'/exp OR 'tamoxifen'/exp OR 'letrozole'/exp OR 'exemestane'/exp OR 'anastrozole'/exp OR 'aromatase inhibitor'/exp 755416

#3 'therapy'/exp OR 'treatment'/exp 10014999

#4 extend OR extended OR 'extension'/exp OR prolonged OR prolongation 1028610

#5 'controlled clinical trial'/exp OR 'randomized controlled trial'/exp 901781

#6 #1 AND #2 AND #3 AND #4 AND #5 389

The Cochrane Library 829

#1 (breast neoplasm) OR (breast tumor) OR (breast cancer) OR (mammary cancer) OR (breast carcinoma) 43503

#2 (hormone) OR (endocrine) OR (anti-hormone) OR (adjuvant) OR (tamoxifen) 89726

#3 (letrozole) OR (exemestane) OR (anastrozole) OR (aromatase inhibitor) 4619

#4 (therapy) OR (treatment) 1109464

#5 (extend) OR (extended) OR (extension) OR (prolonged) OR (prolongation) 87450

#6 (controlled clinical trial) OR (randomized controlled trial) 1314301

#7 #1 AND (#2 OR #3) AND #4 AND #5 AND #6 1065

-Trials matching 829
